# Lectures

**Published:** 1844-09-07

**Authors:** Benjamin Collins Brodie

**Affiliations:** Consulting-Surgeon of the Hospital


### CLINICAL LECTURES AND REPORTS.

### LECTURES

*Delivered in the Theatre of St. George's Hospital, in the session* 1843–44,

BY SIR BENJAMIN COLLINS BRODIE,
Consulting-Surgeon of the Hospital.

*Complicated fistula in ano. Presence of several sinuses. Fistulæ in which the external orifice is not to be found. Fistulæ without external orifice. Fistulæ communicating with the vagina. Fistulæ in perineo.*

Gentlemen,—I concluded the last lecture by speaking to you of the mode of performing the operation for fistula in ano where that fistula is of the simplest kind. But I now come to consider what is to be done in a case of fistula attended with some complication.

The external orifice of the fistula is sometimes at a considerable distance from the verge of the anus, perhaps two or three inches, and in some cases it is as far off even as the buttock. You may, if you please, perform the operation in the same manner. You may pass the probe in at the outer orifice along the fistula into the rectum, feel for the end of the probe in the bowel, and then divide the whole. This, however, is a very serious operation, and a very painful one; you may have considerable hæmorrhage; and under any circumstances there is a very large surface that is to be healed by granulation. But the fact is, this extensive division of parts is really not necessary, and it may be avoided by proceeding in the following manner:—Introduce what I may call the probe-director through the external and internal orifice of the fistula, in the way I have described, and then feel for the probe at some little distance—we will say three-quarters of an inch from the anus. Having felt the probe in that situation, which you may generally do with great ease, with a lancet or double-edged scalpel make an opening through the skin and the adipose substance leading down to the groove of the director. You thus make a new external orifice to the fistula; you then withdraw the probe, pass it into the new orifice you have made, through that into the sinus, and then into the rectum. You then bend the probe, bring out the extremity at the anus, and with a pair of knife-edged scissors, divide the parts that lie over the director, and thus you obtain all that is wanted by a very small division of the soft parts. The fistula is prevented healing by the fæces escaping into it from the rectum and lodging in the narrow channel. Without some such cause as this the whole fistula would heal at once. It is true that the external extremity of the fistula remains undivided, but the fæces cannot pass into it, and in a very short time it heals spontaneously. The internal part is made an open sore: which must be dressed from the bottom, and it heals in the usual manner.

The matter, however, may have burrowed and made many sinuses—a sinus in one direction, and a sinus in another. Sometimes these complicated sinuses are confined to one side of the gut; in other cases they are formed on both sides of it. Before you proceed to the performance of an operation in these cases you must examine the patient very carefully, and it is very probable that three or four examinations will be required before you can ascertain the exact state of the parts sufficiently to guide you in the operation. Introduce the forefinger of the left hand into the gut; then examine the different sinuses, and ascertain whether there is one or more internal communications with the rectum. It very often happens that where there are several sinuses external to the gut, communicating with each other, there is one that is the original sinus, and which has an opening into the bowel. But sometimes there may be a double communication, and then your business is, if possible, to ascertain which is the original sinus, and to lay that open in the way I have already explained, while the others very often need not to be touched at all. If the original sinus be made an open sore the fæces will not pass into the secondary sinuses, and there will be nothing to prevent them from healing.

I have stated that very often it is unnecesary to open more than a single sinus, but there are exceptions to that rule; for there may be sinuses in which the matter lodges, and from which the matter that is formed does not freely escape. These sinuses require to be laid open, not for the purpose of preventing the fæces lodging in them, but on account of the secretion of the sinus itself, just as sinuses anywhere else, from which matter does not freely escape, may require to be freely opened.

I have already stated that if you conduct your examination carefully, and look for the internal orifice of the fistula in the right place, just immediately above the sphincter muscle, you will scarcely ever fail to find it; that if you do not succeed on the first occasion, you will on the second or third. But sometimes the opening is so small, and the sinus takes such a circuitous course, that even after two or three examinations you cannot find it. This will occur sometimes, not very often, and what is then to be done? Perhaps if you were to delay the operation still longer you might discover it, but the patient grows uneasy and impatient at the cure not being completed, and is anxious for something to be done. You must then do what Mr. Pott recommends to be done on all occasions, and which, though a bad practice on all occasions, is a good one sometimes. An artificial opening must then be made into the gut, and you must use the probe-director, or a common probe-pointed bistoury, just as you please. With the forefinger of one hand in the rectum, to assist you, you must, with the instrument, whichever you use, perforate the membrane of the gut some way above the sphincter muscle, and then divide the sinus. But this is, after all, a very unsatisfactory way of doing the operation, and you may rest assured that if you make an artificial opening and fail to find the real and original opening, in three cases out of four you will be plagued afterwards. You have made an artificial opening but the original one remains, and you go on dressing the sore; but there is a little infiltration of fæces and mucus into it that prevents it being healed. When you have to make an artificial opening in the way I have stated I advise you to do something more. Having made the artificial opening, and laid the fistula open into the gut take a straight probe-pointed bistoury, introduce it into the rectum, turn its cutting edge outward, divide the sphincter muscle, and set that completely at liberty. No large division of parts is necessary for this purpose, but having set the sphincter muscle completely at liberty you will scarcely have any trouble in the healing of the sinus. This is better than merely laying open the sinus into the gut where you cannot find the internal orifice; but it is not so good as the operation where you can find it, because you have more bleeding, you give the patient more pain, and there is a large wound to heal. I may, however, take this opportunity of mentioning that although the bleeding from the division of the sphincter muscle is considerable at the time, yet it is never dangerous, because it is within reach. Probably, you may see the vessel that is divided, and can secure it by a ligature; but if not, a dossil of lint dipped in a styptic lotion, laid on the part, and kept there by the finger of an assistant for half an hour, will always stop it.

I mentioned in the last lecture two classes of cases in which the fistula has no external orifice. In one of these there is a small internal opening, and the fæces having penetrated the cellular membrane external to the gut, an abscess has been formed which has burst into the rectum by another opening. In these cases, by making pressure externally, you may generally feel where the matter is lodged. One day the bag is empty, another it is full. Take the opportunity when it is full, and you can feel where it is situated, to make a puncture into it with a lancet, and having so done you reduce it into the state of a common fistula, except that there are two internal openings into it instead of one. You then introduce the probe into the rectum and divide the fistula in the usual manner. You must, if you can, discover both the internal openings, and let them both be included in the incision that you make.

I stated that there was another case in which there was an ulcerated cavity in the neighbourhood of the rectum having no external communication, and where the orifice was originally not like a pin-hole, as in common cases, but sufficiently large to admit the end of the little finger. The ulcer has gone on until it has made a considerable cavity by the side of the gut, having no external opening; and here you are to proceed in the following manner:—The broad internal opening is always close to the sphincter muscle, and at the back part just opposite to the os coccygis. You must be provided with a probe, bent like the one on the table. The probe is to be passed into the rectum, and then drawn down again, so that the point may enter the ulcerated cavity. The point of the probe is felt under the skin; the skin must be punctured with a lancet, and you then introduce the probe-director through the aperture and divide the fistula in the usual manner. [The lecturer illustrated these operations by means of a diagram.]

Now there is another form of fistula of the rectum that requires very special notice. I cannot better explain what I mean than by mentioning the following case:—There was a middle-aged lady who had an abscess formed in front of the rectum. I imagine that it arose, in the usual manner, from ulceration of the gut. The abscess burst close by the posterior margin of the vagina, and appeared just like a common fistula. She consulted a surgeon, who inadvertently treated it as such, and laid it open into the gut. But what was the consequence? He divided both the sphincter ani and the sphincter vagina, and the wound never perfectly healed. She was in the condition of a patient with lacerated perineum, aud all the rest of her life was liable to an involuntary discharge of fæces, of course making her life miserable. I saw this case some twenty-five years ago, and it was, as you may suppose, a lesson to me ever afterwards. It is not very often that abscesses of the rectum do burst in this situation; I have only seen a few examples of it, but the case I have mentioned was sufficient to show me that some peculiar mode of treatment was necessary. How is such a case to be treated? I have seen two or three cases of this kind of fistula since, without having an opportunity of following up the treatment, and no such opportunity occurred till last year. A lady consulted me with a fistula communicating with the rectum in front, and opening externally just at the beginning of the vagina. I merely made a free division of the sphincter muscle on both sides so as to set it completely at liberty. I dressed the cut edges of the sphincter muscle, and it was a good while before it regained its complete usefulness. This was just what I intended. The discharge from the fistula immediately became very much diminished; it continued gradually diminishing, and when I last saw her, which was some few months after the operation, it appeared to me that the fistula was soundly healed. Why is it that the fæces get so readily infiltrated into the internal orifice of the fistula? Because there is an obstruction to their passage occasioned by the sphincter muscle. I divided that muscle, removed that obstruction, and the fæces escaped so easily that they did not get into the internal orifice of the fistula. I was led to adopt this plan of treatment from the course pursued by Mr. Copeland in another case. He says that he was consulted by a lady who had an ulcerated opening between the rectum and vagina. He divided the sphincter muscle, set it completely at liberty, and after the lapse of some time the recto-vaginal communication was closed, and at last firmly cicatrised.

Having stated how these fistulous sinuses are to be laid open, let me say a few words about the dressing. First of all, if the operation be done in a proper manner, there is very little in general to dress—it is only a narrow sore that remains to be dressed. Do not cram it with lint; all that is necessary is, to put a little lint between the edges to prevent them prematurely healing. The parts about the rectum are very often a little longer in healing, and it may be worth while to dress them with red precipitate ointment. When the parts are beginning to granulate you may hasten their cicatrisations and the formation of new skin, by touching them lightly over with the nitrate of silver. It is very seldom necessary, except in complicated cases, to dress the fistula for any length of time; a few days' dressing is very often quite sufficient. As soon as the cut edges are skinned over the dressing is hardly necessary, and it will save both you and the patient a good deal of trouble merely to touch the surface of the sore lightly every other day with the nitrate of silver. When the edges are fairly skinned over, the rest will skin over sooner without the dressing than with it. If you cram the part full of lint you occasion the patient a great deal of pain. I am sure that sometimes, from too much lint being crammed in, the matter does not freely escape; it burrows in the cellular membrane, and makes a fresh sinus.

There are some cases in which abscesses occur about the rectum, which may be confounded with that particular disease I have just described, and I shall explain them in order that you may draw the distinction between them. An abscess sometimes forms in an external pile. The patient has an external pile; it inflames and suppurates, and on going to him you find the abscess just on the point of bursting. You open it and let out perhaps a teaspoonful or more of matter, but on passing in a probe it will not go up by the side of the gut. This is a very troublesome sort of abscess, it is very painful, the patient ean hardly bear to go to the water-closet, and he has pain in passing the last drops of urine.

The treatment is very simple. You cure it at once radically by snipping off the external pile, abscess and all, with a pair of curved scissors.

The same thing will sometimes happen with an internal pile. The patient has an internal pile, inflammation takes place in it, an abscess forms and bursts externally, and you can pass a probe into the abscess in the inside of the pile. Here, also, the best way is, if the pile be small, to snip it off with a pair of scissors, or if it be not small to tie it with a silk thread round the base, and destroy it by ligature. I may here mention an error into which you will be liable to fall if you be not on your guard against it. When you introduce a probe into an abscess formed in an internal pile it very easily breaks down the slender wall of the abscess, and runs into the cellular substance under the mucous membrane. The cellular tissue offers so little resistance to the probe that it may pass in any number of inches between the mucous membrane and the muscular tunic without your being aware of the circumstance. I remember a case many years ago where a surgeon of great eminence in this town laid open what he thought was a sinus of two or three inches in length into the rectum. I am satisfied, from what I remember of the case and have since seen, that it was an abscess formed in an internal pile, and that what he supposed to be a sinus was neither more nor less than a space he had made himself by running the probe into the loose cellular texture.

It is necessary, in the very great majority of cases, to lay the kind of sinuses to which I have alluded completely open into the rectum; and I presume that it is from the analogy to fistula here that some surgeons have been led to think that this operation is necessary for all kinds of fistulous sinuses. I remember some very good surgeons in this town who used to think it was requisite to open what is termed a fistula in perineo in this manner. There can be no greater error. A fistula in perineo is the same as a fistula in ano, except that it communicates with the urethra behind a stricture, whereas a fistula in ano communicates with the rectum above the sphincter muscle. The fistula in perineo is the result of some of the urine passing in from the urethra, and to lay it open will do no good, for it will not prevent the escape of urine going on. But this may be accomplished by dilating the stricture, and, in nineteen cases out of twenty, all that you have to do is, to dilate the stricture. Generally, by the time the stricture is dilated, the urine, finding a readier passage forward than it does through the ulcerated opening, will not pass into the latter, and the fistula is usually healed by the time the stricture is dilated. If it be not completely healed by that time you have only to keep the stricture dilated for a considerable period by the introduction of an instrument every day, or every other day, and the fistula in perineo will at last heal. If it be a large opening it will take some months to heal, but still it heals spontanously. There is only one kind of case in which it is necessary to lay open a fistula in perineo, and that is, where there is a sinus in the perineum into which the urine escapes, but which is so situated that neither the urine nor the matter secreted in the sinus can find egress. If there be a fistula in perineo under these circumstances it may require to be opened.

There are some fistulous sinuses that exist in the groin in connection with disease in the glands of the groin. Surgeons formerly supposed that these required to be laid open like a fistula in ano. They do require to be opened where matter lodges in them and cannot escape, or, at any rate, a counter-opening will be necessary; for there is no disposition to heal unless the matter escapes as fast as it is secreted; but the mere laying open of the fistula will not cause it to heal, it will only prevent it extending. What hinders the fistula in the groin from healing? The diseased gland at the bottom of it. If you wish the fistula to heal you must destroy the diseased gland, or bring it into a healthy condition. Sometimes it may be necessary to dissect out the gland or to destroy it by a powerful escharotic; but in the greater number of cases, if you attend to the general health, the diseased gland recovers itself, and so soon, and no sooner, will the sinus in the groin heal.

The same observation applies to fistulæ that are connected with dead bone. A fistulous sinus leading down to dead bone does not heal because there is dead bone in the bottom; but if the dead bone comes away then the fistula will heal. It is needless to lay open the fistula to inject stimulating liquors into it, or to do anything till the dead bone has been removed. All that it is worth while to do is, if matter lodges in it to make a counter-opening by which it may escape.—*London Lancet.*

